# Intracranial Aneurysm Risk Locus 5q23.2 Is Associated with Elevated Systolic Blood Pressure

**DOI:** 10.1371/journal.pgen.1002563

**Published:** 2012-03-15

**Authors:** Emília Ilona Gaál, Perttu Salo, Kati Kristiansson, Karola Rehnström, Johannes Kettunen, Antti-Pekka Sarin, Mika Niemelä, Antti Jula, Olli T. Raitakari, Terho Lehtimäki, Johan G. Eriksson, Elisabeth Widen, Murat Günel, Mitja Kurki, Mikael von und zu Fraunberg, Juha E. Jääskeläinen, Juha Hernesniemi, Marjo-Riitta Järvelin, Anneli Pouta, The International Consortium for Blood Pressure Genome-Wide Association Studies (ICBP-GWAS), Christopher Newton-Cheh, Veikko Salomaa, Aarno Palotie, Markus Perola

**Affiliations:** 1 Public Health Genomics Unit, Department of Chronic Disease Prevention, National Institute for Health and Welfare, Helsinki, Finland; 2 Institute for Molecular Medicine Finland (FIMM), University of Helsinki, Helsinki, Finland; 3 Department of Neurosurgery, Helsinki University Central Hospital, Helsinki, Finland; 4 The Wellcome Trust Sanger Institute, Hinxton, United Kingdom; 5 Research Centre of Applied and Preventive Cardiovascular Medicine, University of Turku, Turku, Finland; 6 Department of Clinical Physiology, University of Turku and Turku University Hospital, Turku, Finland; 7 Department of Clinical Chemistry, University of Tampere and Tampere University Hospital, Tampere, Finland; 8 Diabetes Unit, Department of Chronic Disease Prevention, National Institute for Health and Welfare, Helsinki, Finland; 9 Department of General Practice and Primary Health Care, Institute of Clinical Medicine, University of Helsinki, Helsinki, Finland; 10 Vasa Central Hospital, Vasa, Finland; 11 Folkhälsan Research Centre, Helsinki, Finland; 12 Unit of General Practice, Helsinki University Central Hospital, Helsinki, Finland; 13 Department of Neurosurgery, Yale University School of Medicine, New Haven, Connecticut, United States of America; 14 Neurosurgery of NeuroCenter, Kuopio University Hospital, Kuopio, Finland; 15 Department of Biostatistics and Epidemiology, School of Public Health, Faculty of Medicine, Imperial College, London, United Kingdom; 16 Institute of Health Sciences, University of Oulu, Oulu, Finland; 17 Biocenter Oulu, University of Oulu, Oulu, Finland; 18 Department of Children, Young People and Families, National Institute for Health and Welfare, Oulu, Finland; 19 Center for Human Genetic Research, Cardiovascular Research Center, Massachusetts General Hospital, Boston, Massachusetts, United States of America; 20 Program in Medical and Population Genetics, Broad Institute of Harvard and MIT, Cambridge, Massachusetts, United States of America; 21 Chronic Disease Epidemiology and Prevention Unit, Department of Chronic Disease Prevention, National Institute for Health and Welfare, Helsinki, Finland; 22 Program in Medical and Population Genetics and Genetic Analysis Platform, Broad Institute of Harvard and MIT, Cambridge, Massachusetts, United States of America; 23 Department of Medical Genetics, University of Helsinki and University Central Hospital, Helsinki, Finland; 24 University of Tartu, Estonian Genome Centre, Tartu, Estonia; University of Miami, United States of America

## Abstract

Although genome-wide association studies (GWAS) have identified hundreds of complex trait loci, the pathomechanisms of most remain elusive. Studying the genetics of risk factors predisposing to disease is an attractive approach to identify targets for functional studies. Intracranial aneurysms (IA) are rupture-prone pouches at cerebral artery branching sites. IA is a complex disease for which GWAS have identified five loci with strong association and a further 14 loci with suggestive association. To decipher potential underlying disease mechanisms, we tested whether there are IA loci that convey their effect through elevating blood pressure (BP), a strong risk factor of IA. We performed a meta-analysis of four population-based Finnish cohorts (n_FIN_ = 11 266) not selected for IA, to assess the association of previously identified IA candidate loci (n = 19) with BP. We defined systolic BP (SBP), diastolic BP, mean arterial pressure, and pulse pressure as quantitative outcome variables. The most significant result was further tested for association in the ICBP-GWAS cohort of 200 000 individuals. We found that the suggestive IA locus at 5q23.2 in *PRDM6* was significantly associated with SBP in individuals of European descent (p_FIN_ = 3.01E-05, p_ICBP-GWAS_ = 0.0007, p_ALL_ = 8.13E-07). The risk allele of IA was associated with higher SBP. *PRDM6* encodes a protein predominantly expressed in vascular smooth muscle cells. Our study connects a complex disease (IA) locus with a common risk factor for the disease (SBP). We hypothesize that common variants in *PRDM6* can contribute to altered vascular wall structure, hence increasing SBP and predisposing to IA. True positive associations often fail to reach genome-wide significance in GWAS. Our findings show that analysis of traditional risk factors as intermediate phenotypes is an effective tool for deciphering hidden heritability. Further, we demonstrate that common disease loci identified in a population isolate may bear wider significance.

## Author Summary

When multiple genes or genetic regions contribute to the inherited risk of a disease, it is referred to as a complex disease. Genome-wide association studies (GWAS) aim to detect common genetic variations that associate with complex traits or diseases. Although GWAS have been successful in identifying strongly associated genetic loci, they lack the means to point out true, but less strong, associations. Studying conditions that are related to the disease of interest can help sort out less strong associations. Intracranial aneurysms (IA) are berry-like dilations in cerebral arteries. Most IAs do not give symptoms until they bleed, causing a highly fatal form of stroke. Half of the people who suffer bleeding of an IA die. IA is a complex disease. Both inherited risk and environmental factors contribute to the risk of developing IA. Women, smokers, those with high alcohol intake or high blood pressure are more prone to develop IA and bleeding. GWAS found 19 genetic regions increasing the risk of IA. Here we show that one of these loci, on the long arm of chromosome 5, in addition to raising IA risk also increases systolic blood pressure. We speculate that the cause is modified vascular wall structure.

## Introduction

Intracranial aneurysms (IA) are berry-shaped pouches at the branching sites of cerebral arteries. 2–5% of the world population is estimated to harbor IA [1]. Most IA go unnoticed during one's lifetime. However, when they become symptomatic, it is usually due to rupture, causing subarachnoid hemorrhage (SAH). SAH is devastating intracranial bleeding, and half of those with SAH die within a year [2], [3]. SAH affects the working age population, with a median age of 55 [4]. Its incidence in Finland is 19/100 000/year [5], [6], triple than that of the rest of the world. The reason for this higher than average incidence is unknown. Aneurysmal SAH places a heavy burden on society both emotionally and financially. The strongest known non-modifiable risk factor of SAH is family history of the disease, and the strongest modifiable risk factors are smoking, excessive alcohol intake, and hypertension [7]. An important step in tackling SAH is to understand why IAs develop.

Our understanding of the environmental and genetic background of IA formation is limited. Positive family history of IA or SAH, older age and female sex increase the risk of developing IA [1]. Of the general cardiovascular risk factors, smoking has been shown to increase the risk of IA formation [8], and high blood pressure has long been speculated to do so [9]. The high, often undocumented, prevalence of high blood pressure in the control populations is likely the reason why it frequently fails to reach statistical significance as an IA risk factor [1]. Chronic hypertension may contribute to IA formation by imposing constantly high shear stress on vascular walls [9].

Multiple factors, such as familial aggregation of the disease, make a genetic contribution likely to the risk of IA. A minority of IAs show familial aggregation (under 10%) [7]. Linkage studies in IA families have highlighted numerous genetic regions and a recent exome sequencing study identified coding mutations in familial thoracic aortic aneurysm with intracranial aneurysm [10]. However, the majority of IA is sporadic. Sporadic IA is a complex disease and no gene with a certain role has been identified yet. Recent genome-wide association studies (GWAS) [11], [12] involving Finnish IA patients, have attempted to decipher the complex genetic background of IA. From these studies, five loci emerged with strong association to IA (p<5E-07, posterior probability of association –PPA>0.5), with the highest statistical significance at 9p21.3, a risk locus of multiple cardiovascular diseases. Further 14 loci exhibited suggestive association to IA (0.1≤PPA<0.5).

Despite the success of GWAS in identifying IA susceptibility loci, the pathomechanism by which they contribute to IA formation remains elusive. We hypothesize that hypertension, a strong modifiable risk factor of IA, may possess an overlapping genetic background with IA. To test this hypothesis, we analyzed the IA loci so far identified, in well-characterized population-based cohorts consisting of more than 210 000 individuals with blood pressure measurements.

## Results

41 SNPs from 19 independent IA loci [13] were first analyzed for association with blood pressure in the national Health 2000 survey (H2000) [14] discovery cohort of 1581 individuals without blood pressure lowering medication (Table S1). We adjusted the analysis for age and gender (ROBUST model). The most significant association (p<0.1) were observed at 2q33.1 with diastolic blood pressure (DBP) and with mean arterial pressure (MAP), at 4q31.23 and 19q13.12 with DBP, and at 5q23.2 with systolic blood pressure (SBP), DBP, and MAP. We did not detect association with pulse pressure (PP) (Table S1). Next, we wanted to analyze the independence of the association signals observed. We tested all 19 loci SNPs adjusting for further factors known to affect blood pressure, namely smoking habits, alcohol consumption, and body mass index (BMI) (ADVANCED model). There was no tendency of association with DBP at 4q31.23 and 19q13.12 with the ADVANCED model. The strength of the association decreased for the four SNPs at the 2q33.1 locus for SBP, but increased marginally for DBP, and MAP. At 5q23.2 the strength of association increased substantially for most blood pressure measurements, such as SBP, DBP, and MAP (Table 1 and Table S2) for all three SNPs tested. The IA risk alleles at 5q23.2 were associated with elevated blood pressure.

**Table 1:** 2q33.1 and 5q23.2 loci cohort-wise ADVANCED model effect estimates and meta-analysis results with systolic blood pressure (SBP).$

				Discovery - beta (SE)	Replication - beta (SE)			Meta SBP*	
Locus	SNP	IA Risk Allele	MAF	H2000	YFS	NFBC1966	HBCS	p	beta (SE)
2q33.1	rs1429412	G	0.5	0.75 (0.64)	−0.27 (0.42)	−0.15 (0.24)	0.05 (0.85)	6.65E-01	−0.08 (0.19)
2q33.1	rs12472355	A	0.5	0.73 (0.64)	−0.24 (0.42)	−0.11 (0.24)	−0.03 (0.85)	7.71E-01	−0.06 (0.19)
2q33.1	rs787997	A	0.4	0.62 (0.65)	−0.11 (0.42)	−0.08 (0.24)	0.25 (0.84)	9.66E-01	−0.01 (0.19)
2q33.1	rs787994	T	0.4	0.68 (0.65)	0.03 (0.42)	−0.03 (0.24)	0.26 (0.85)	7.53E-01	0.06 (0.19)
5q23.2	rs570682	T	0.2	1.48 (0.69)	1.22 (0.47)	0.71 (0.27)	0.46 (0.98)	4.80E-05	0.87 (0.22)
5q23.2	rs2287696	A	0.2	1.68 (0.71)	1.18 (0.49)	0.67 (0.28)	0.93 (1.03)	6.81E-05	0.89 (0.22)
5q23.2	rs335206	C	0.4	1.02 (0.60)	0.85 (0.40)	0.74 (0.24)	0.60 (0.84)	3.01E-05	0.79 (0.19)

Genomic positions are based on the human genome build 36. Alleles are reported on the forward strand of the reference genome. The effects are reported for the alleles increasing risk for IA in the Yasuno et al. studies [12], [13]. Risk alleles are aligned according to the forward strand of the reference genome. Minor allele frequencies (MAF) are based on from the HapMap Phase II CEU population data.

$ Diastolic blood pressure (DBP) and mean arterial pressure (MAP) association results from 2q33.1 and 5q23.2 SNP are in Table S2.

*: Meta SBP: meta-analysis of discovery and replication cohort p-values and beta for systolic blood pressure (SBP) with the ADVANCED model. Association analyses were corrected for gender, age, BMI, smoking habits and alcohol consumption.

SE: standard error.

**Figure 1:**
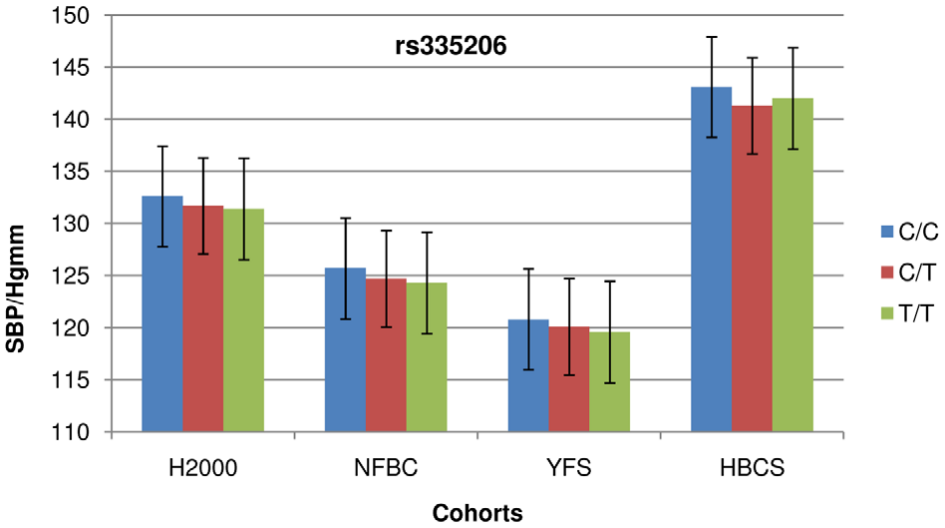
Cohort-wise effects of risk allele count on SBP. The higher median age in HBCS is reflected as higher systolic blood pressure (SBP) and less consistent association. Error bars show standard error.

To confirm the initial association signals at the 2q33.1 and 5q23.2 loci observed in the H2000 discovery cohort, we tested them for association with blood pressure in three additional population-based cohorts from Finland. SNPs at 2q33.1 failed to show significant association with DBP and MAP in any of the replication cohorts (the Cardiovascular Risk in Young Finns Study-YFS [15], [16], the Northern Finland Birth Cohort 1966-NFBC1966 [17], and the Helsinki Birth Cohort Study-HBCS [18]). When the results were combined from all cohorts in a fixed effect meta-analysis, they remained non-significant (Table 2). At 5q23.2 SNPs showed significant association with SBP in YFS and NFBC1966 (Table 1). In HBCS, although consistent in the direction of the effect, the association remained suggestive. When the results were combined from all cohorts in a fixed effect meta-analysis, we detected significant association with SBP at 5q23.2 (p_rs570682_ = 4.80E-05, p_rs2287696_ = 6.81E-05, p_rs335206_ = 3.01E-05) (Table 1). Comparing the mean SBP of the study participants stratified for their 5q.23.2 genotypes indicated a positive correlation between the number of risk alleles and higher SBP for all three SNPs tested (Figure 1). Study participants homozygous for the risk allele (*C*, in the case of rs335206), had on average 1.3 Hgmm higher SBP compared to those who were homozygous for the protective allele, and 0.9 Hgmm higher than those with the heterozygous genotype. This effect size is comparable to those of most blood pressure loci identified by The International Consortium for Blood Pressure Genome-wide Association Studies (ICBP-GWAS) consortium [19]. The observed linear effect of risk allele count is strongly suggestive of a true association. Association at 5q23.2 with DBP (p_rs570682_ = 0.02, p_rs2287696_ = 0.04, p_rs335206_ = 0.03) and MAP (p_rs570682_ = 0.0007, p_rs2287696_ = 0.0010, p_rs335206_ = 0.0004) showed a reduction of significance when results were combined from all cohorts (Table S2).

**Table 2:** Summary of leading SNPs from the 19 loci showing strong or suggestive association with IA in a multinational GWAS containing Finnish patients [12].

*Representative SNPs of IA loci with PPA>0.5 (Yasuno et al 2010 * [*12*] *)*											
				IA GWAS (Yasuno et al. 2010)#				SBP meta-analysis with ROBUST model$		SBP meta-analysis with ADVANCED model$	
Locus	SNP	Position	Gene	Risk Allele	Finnish p	OR (95% CI)	PPA##	beta (SE)	p	beta (SE)	p
8q12.1	rs9298506*	55600077	3′-SOX17	A	1.00E-05	1.39(1.20–1.61)	0.9999	−0.03 (0.23)	8.80E-01	−0.09 (0.22)	6.79E-01
9p21.3	rs1333040	22073404	CDKN2A/B	T	5.30E-08	1.39(1.23–1.56)	0.9999	0.07 (0.19)	7.20E-01	−0.04 (0.19)	8.24E-01
10q24.32	rs12413409	104709086	CNNM2	G	4.20E-02	1.27(1.01–1.59)	0.9990	0.62 (0.35)	7.78E-02	0.67 (0.34)	4.99E-02
13q13.1	rs9315204	32591837	STARD13	T	1.70E-04	1.27(1.12–1.44)	0.9981	0.04 (0.21)	8.64E-01	0.12 (0.20)	5.51E-01
18q11.2	rs11661542	18477693	RBBP8	C	2.30E-02	1.14(1.02–1.28)	0.9999	0.00 (0.20)	9.81E-01	0.00 (0.19)	9.86E-01

*Representative SNPs of IA loci with 0.1≤PPA<0.5 (Yasuno et al 2011 * [*13*] *)*											
				IA GWAS (Yasuno et al. 2010)#				SBP meta-analysis with ROBUST model$		SBP meta-analysis with ADVANCED model$	
Locus	SNP	Position	Gene	Risk Allele	Finnish p	OR (95% CI)	PPA##	beta (SE)	p	beta (SE)	p
1p36.31	rs1876848	6876262	CAMTA1	G	1.30E-01	1.12 (0.97–1.30)	0.1128	0.03 (0.24)	8.98E-01	−0.09 (0.23)	6.87E-01
1p22.2	rs1725390	91031160	BARHL2-ZNF644	A	1.40E-03	1.21 (1.08–1.36)	0.1011	−0.08 (0.20)	6.72E-01	−0.13 (0.19)	4.92E-01
1q21.3	rs905938	153258013	DCST2	T	6.70E-03	1.22 (1.06–1.41)	0.1252	0.38 (0.23)	9.96E-02	0.37 (0.22)	9.91E-02
2q33.1	rs787994	197931366	ANKRD44-SF3B1	T	2.90E-04	1.25 (1.11–1.41)	0.0988	0.25 (0.20)	2.11E-01	−0.06 (0.19)	7.53E-01
4q31.23	rs6841581*	148620640	upstream EDNRA	G	4.00E-03	1.31 (1.09–1.58)	0.1750	0.11 (0.28)	6.91E-01	0.16 (0.27)	5.54E-01
**5q23.2**	**rs2287696**	**122488231**	**PRDM6**	**A**	**3.60E-04**	**1.28 (1.12–1.46)**	**0.1760**	**0.87 (0.23)**	**1.83E-04**	**0.89 (0.22)**	**6.81E-05**
8p23.2	rs2045637	2963188	CSMD1	A	9.20E-05	1.37 (1.17–1.61)	0.2139	0.31 (0.28)	2.61E-01	0.35 (0.27)	1.95E-01
8q24.23	rs6577930**	139582362	FAM135B-COL22A1	T	3.00E-03	1.21 (1.11–1.33)	0.1386	−0.01 (0.02)	4.84E-01	−0.01 (0.02)	4.58E-01
11q22.2	rs2124216	101644113	YAP1-BIRC3	A	4.50E-04	1.26 (1.11–1.43)	0.1963	0.12 (0.23)	6.00E-01	0.11 (0.23)	6.24E-01
12p13.31	rs728342	5577633	TMEM16B	G	4.80E-02	1.14 (1.00–1.30)	0.1601	−0.14 (0.22)	5.10E-01	−0.18 (0.21)	4.08E-01
12q22	rs6538595	94030754	FGD6	A	1.70E-02	1.17 (1.03–1.34)	0.1136	−0.37 (0.22)	9.15E-02	−0.45 (0.21)	3.68E-02
19q13.12	rs1688005	40340205	FXYD5	G	3.80E-02	1.14 (1.01–1.29)	0.1244	0.17 (0.21)	4.27E-01	0.10 (0.20)	6.34E-01
20p12.1	rs1132274**	17544155	RRBP1	A	1.00E-02	1.23 (1.05–1.45)	0.1435	0.04 (0.02)	2.69E-02	0.03 (0.02)	1.37E-01
22q12.1	rs133885	24489289	MYO18B	G	2.20E-01	1.08 (0.96–1.21)	0.1230	0.19 (0.19)	3.18E-01	0.30 (0.19)	1.16E-01

Association results with intracranial aneurysm (IA) by Yasuno and colleagues are followed by our meta-analysis association results with systolic blood pressure (SBP), with the ROBUST and the ADVANCED models, respectively.

Table first shows association p-values with IA for the Finnish sub-group from the multinational GWAS (IA GWAS), followed by results from our meta-analysis of association with systolic blood pressure (SBP) with the ROBUST and ADVANCED models. In the ROBUST model of association we corrected for gender and age and in the ADVANCED model we further corrected for BMI, smoking habits and alcohol consumption.

Genomic positions are based on the human genome build 36. Alleles are reported on the forward strand of the reference genome. The effects are reported for the alleles increasing risk for IA in the Yasuno et al. studies [12], [13]. If SNP is intergenic, Gene represents the nearest gene. SNPs are directly genotyped unless otherwise marked (* HM2 imputed SNP, ** 1000G+HM3 imputed SNP). Yasuno et al (2011) at 8q24.23 followed-up with rs1554349 instead of the lead SNP, rs6577930.

**In bold:** locus showing strongest association with SBP in meta-analysis.

# ‘IA GWAS’ triplet column shows the Finnish sub-group (n_FINN-IA-CASES_ = 912, n_FINN-CONTROLS_ = 8180) association results on IA of the GWAS by Yasuno and colleagues, except for the PPA results, which is not Finnish sub-group specific, but counted for the whole multinational cohort.

## PPA: posterior probability of association with IA as calculated by Yasuno and colleagues for the multinational IA GWAS.

$ ‘SBP meta-analysis with ROBUST model’ and ‘with ADVANCED model’ twin-columns show results of our candidate locus meta-analysis with SBP as the outcome variable. SBP meta-analysis beta values are given for IA risk alleles.

OR: odds ratio, CI: confidence interval, SE: standard error.

To test whether the association at the 5q23.2 locus is unique to the Finnish cohorts, we attempted to replicate the association with the three SNPs in the multinational cohort ICBP-GWAS [19]. All three SNPs showed significant association with SBP (p_rs570682_ = 0.0065, p_rs2287696_ = 0.00079, p_rs335206_ = 0.0014) in the ICBP-GWAS cohort of 200 000 individuals of European descent. The risk allele for elevated SBP in the ICBP-GWAS cohort was the same as in our meta-analysis of four Finnish population-based cohorts. When the results from the four Finnish cohorts were combined with the ICBP-GWAS results in a fixed effect meta-analysis, the strength of the association increased with all three SNPs tested (Table 3). The strongest association was observed with rs2287696 (p_ALL_ = 8.13E-07). This suggests that the variant at 5q23.2 is a common risk factor present in multiple populations of European descent. Further loci or results for DBP or MAP were not tested for association in ICBP-GWAS, since they failed to show significant association in our replication cohorts.

**Table 3:** Meta-analysis results of 5q23.2 SNPs with systolic blood pressure from all four Finnish cohorts and ICBP-GWAS combined.

SNP	Position	Risk Allele	beta (SE)	p
rs570682	122477549	T	0.50 (0.11)	9.58E-06
rs2287696	122488231	A	0.57 (0.16)	8.13E-07
rs335206	122532465	C	0.41 (0.86)	2.00E-06

Genomic positions are based on the human genome build 36. Risk alleles are aligned according to the forward strand of the reference genome. SE: standard error.

**Figure 2:**
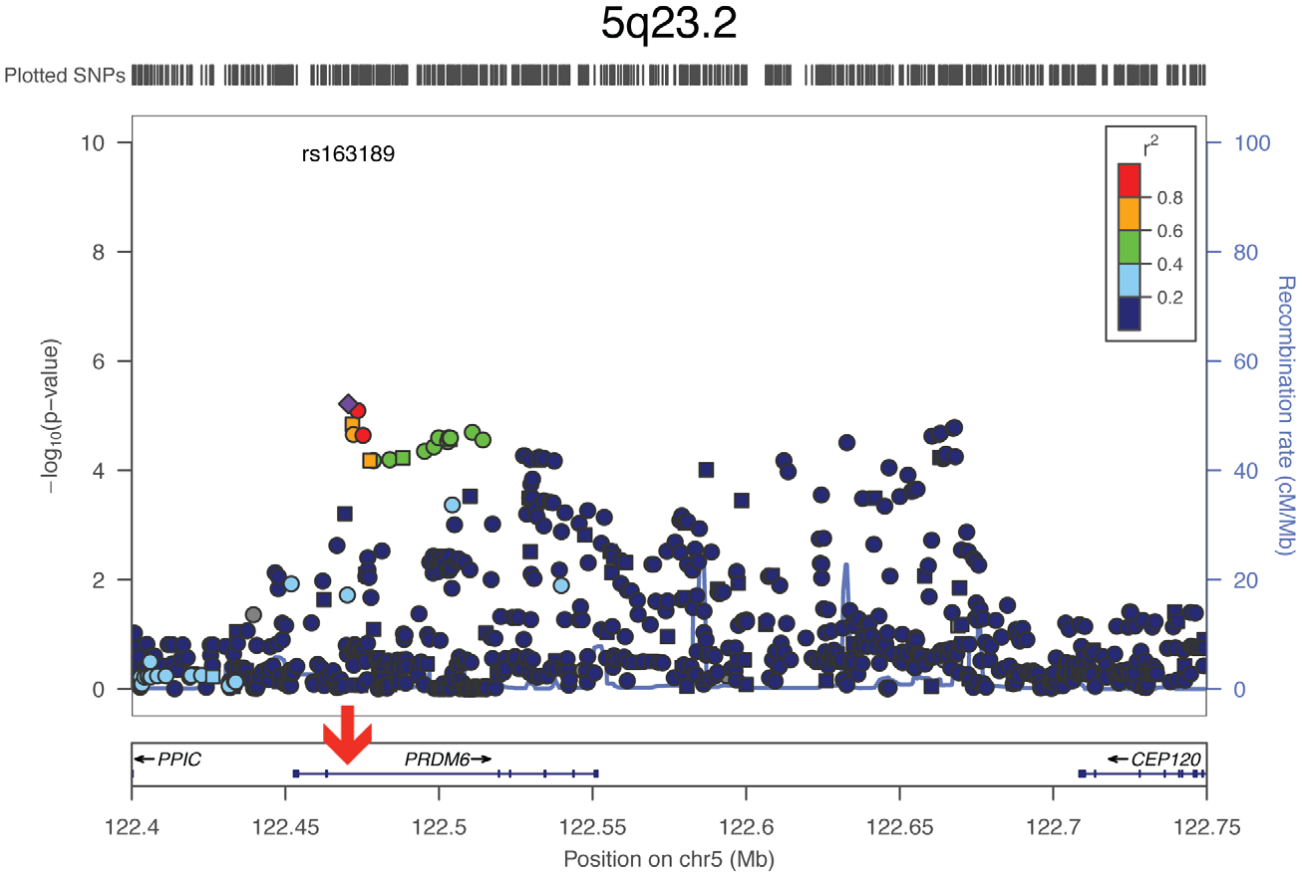
Association with SBP around PRDM6: Meta-analysis results from the four Finnish cohorts. Square shapes represent genotyped SNPs, circles imputed ones. Red arrow depicts the SREBP1 binding site. For each SNP, the symbol color indicates the SNP's pairwise linkage disequilibrium with the most significant SNP, rs163189, measured as r2. SNPs for which no recombination data is available from the 1000 Genomes June 2010 CEU panel are in grey.

All three tested SNPs at 5q23.2 reside in intronic regions of the gene *PR domain containing 6* (short form: *PRDM6*) and showed comparable p-values. To further explore the associated region in an attempt to pinpoint the causative variant, we examined all 1000 Genomes variants around *PRDM6* in the four Finnish cohorts (Figure 2). The strongest association was observed with rs163189 (p = 6.12E-06) near rs570682 and rs2287696, in the second intron, where the most significantly associated SNPs clustered. All five of the strongest associated SNPs are located within a 4.7 kb region at 122.4 MB (Human genome build 36), surrounding a Sterol regulatory element binding transcription factor 1 (SREBP1) binding site (Figure 2) [20].

## Discussion

Hypertension, a leading cardiovascular risk factor, is a strong modifiable risk factor for IA and its deadly rupture. Our study establishes a genetic link between elevated SBP and IA formation. Further, we demonstrate the benefits of using population isolates for mapping complex disease loci valid in multiple populations.

5q23.2 was identified as a suggestive IA risk locus by Yasuno and colleagues [13] in a multinational GWAS including Finnish IA patients. The strength of the association at 5q23.2 in their study mainly came from the Finnish cohort (Figure S1). However, albeit weaker, association to IA at 5q23.2 was observable in all cohorts tested by Yasuno and colleagues. In the two tier approach we applied, the suggestive aneurysmal locus at 5q23.2 showed robust association to blood pressure traits in three cohorts (namely the discovery cohort H2000, and the replication cohorts NFBC1966 and YFS). The trend of the effect was the same while the association remained suggestive with blood pressure traits in the HBCS. HBCS participants' average age was higher (61 years) than that of the rest (36 years) (Figure S2). With age, the relative contribution of genetic predisposition and lifestyle may change, potentially accounting for the less significant association in HBCS.

In our meta-analysis of candidate loci the most significant association was observed at 5q23.2 in *PRDM6*. Although an association can be observed throughout the whole gene, fine-mapping of the region with 1000 Genomes variants revealed the focus of association to be within a 4.7 kb region in the second intron (Figure 2). *PRDM6* encodes an epigenetic modulator of transcription with roles in endothelial [21] and vascular smooth muscle cells (SMC) [22]. PRDM6 has a critical role in arterial wall SMC, where it is predominantly expressed. PRDM6 participates in the phenotypic switch between proliferative and differentiating vascular SMC phenotypes [22]; when active, PRDM6 inhibits differentiation and promotes proliferation. Excess vascular SMC proliferation is an important pathomechanism in hypertension, and it exacerbates the vascular wall remodeling often seen in IA [23], [24]. When vascular SMCs re-enter the cell cycle to proliferate, they lose their contractile qualities. Distinct from extracranial arteries, cerebral arteries lack an external elastic lamina and the adventitia is weakly developed, making them inflexible, and less resistant to stress [25]. It is possible that when SMC proliferation further stiffens cerebral arteries, they become incapable of adjusting to shear stress, and give way to IA formation. This is a plausible explanation to why the intracranial manifestation of a supposedly generalized vasculopathy can be so distinct. Intriguingly, excessive vascular SMC proliferation is part of the pathomechanism of the strongest common IA risk locus at 9p21.3 [26]. However, to test possible causality, examination of whether the risk variant at 5q23.2 is associated with higher PRDM6 activity is necessary. Although the causative variant remains elusive, we succeeded in narrowing down the associated region markedly. The 4.7 kb region showing the strongest association harbors a SREBP1 binding site. SREBP1 is a transcription factor governing cellular lipid biosynthesis. Highlighting its biological significance in vascular traits, non-synonymous mutations in SREBP1 cause spontaneous hypertension in rats [27]. It is possible that common variants facilitate SREBP1 binding, and thus, as shown by Zhou and colleagues [28], cause vascular SMC proliferation. We propose that this effect is conveyed by PRDM6 activation.

Although both the location and the function of the gene highlight *PRDM6* as a likely candidate, it is not the only plausible gene near the association signal. *Centrosomal protein of 120 KD* (short form: *Cep120*) is just downstream from the region of association (Figure 2). *Cep120* is a centrosomal protein with preferentially high expression in neuronal progenitors during development [29]. *Cep120* could contribute to IA risk by causing perturbation in the neurovascular niche.

This is the first study establishing a shared genetic background at 5q23.2 for IA and its important risk factor, high blood pressure. However, both IA [30], [31] and hypertension [32] have shown linkage to 5q23.2 in previous studies. Resequencing the genomic region in families that previously showed linkage to 5q23.2 might reveal penetrant variants causing familial IA or severe high blood pressure, or possibly both. Notably, Vasan and colleagues [33] found that rs17470137, less than 8 kb downstream from *PRDM6*, is associated with aortic root size, a feasible proxy of blood pressure [34].

GWAS are designed to identify associations, they do not prove causality. Deep resequencing of the associated region may improve the fine mapping and guide closer to the causative variant, or even uncover it, although resequencing efforts of GWAS regions have had limited success [35]. A further limitation of our study is that we were unable to address whether the identified risk variant at 5q23.2 increases the risk of developing IA as a consequence of elevated SBP (causality between high SBP and IA) or whether the variant modifies vessel wall structure in a way that elevates SBP and increases IA risk as a pleiotropic effect (Figure S3). A study conducted in a cohort characterized both for IA and blood pressure would likely be a more suitable way of addressing this question. Unfortunately, to the best of our knowledge, such a large-scale cohort does not currently exist. The identified risk variant, however, is unlikely to confer its effect solely by increasing blood pressure, as leading hypertension risk loci fail to show association with IA (data not shown). Yet, the mechanical effect of elevated BP on the vessel wall, likely exacerbates IA formation. The significance of the association identified in our study awaits confirmation in other ethnicities.

To further decipher the genetics of IA, it is important to test if genetic links can be established between IA and other strong risk factors, such as smoking and alcohol consumption. In conclusion, our results highlight the link between IA and blood pressure.

## Materials and Methods

### Study Subjects

Four Finnish population-based cohorts were included in our study (Table 4). These cohorts were not characterized for IA. We utilized genome-wide genotyped participants with available blood pressure data, excluding those on blood pressure medication and those for whom blood pressure medication data was not available (n_excluded_ = 1373). In our two tier approach, the discovery cohort (n_discovery_ = 1581) was a subsample of the H2000 [14]. The H2000 study was carried out in several regions of Finland from fall 2000 to spring 2001, and was designed to provide information on the health of the Finnish population. A subset of this cohort, consisting of metabolic syndrome cases and matched controls, was genotyped and utilized in this analysis. The replication cohort (n_replication_ = 8312) consisted of the YFS (n = 1874) [15], [16], the NFBC1966 (n = 5361) [17], and the HBCS (n = 1077) [18]. YFS participants were recruited from all around Finland for a large follow-up study on cardiovascular risk factors in young individuals in 1980. Clinical data are from the follow-up at age 27 performed in 2007. NFBC1966 comprises individuals born in 1966 in the two northernmost provinces of Finland (Oulu and Lapland). Clinical examinations took place at the follow-up at age 31 in 1997. The HBCS participants were recruited from the Helsinki region. The study examines the impact of fetal environmental factors on childhood and adult life. Clinical examinations took place during 2001–2004. HBCS participants had the highest average age (Figure S2).

**Table 4:** Summary of cohort characteristics.

	*Discovery*	*Replication*			*Meta*
Characteristic	H2000	YFS	NFBC1966	HBCS	TOTAL
WG genotyped, QC passed	2210	2019	5361	1676	11266
WG genotyped, QC passed NOT taking BP medications	1581	1874	5361	1077	9893
Included in ROBUST model analysis	1575	1855	5242	1043	9715
Included in ADVANCED model analysis	1575	1805	5031	1038	9449
SBP (Hgmm, mean (SD))	132 (19)	120 (14)	125 (13)	142 (20)	127 (17)
DBP (Hgmm, mean (SD))	83 (11)	75 (11)	77 (12)	88 (10)	79 (12)
MAP (Hgmm, mean (SD))	107 (13)	98 (12)	101 (11)	115 (14)	103 (13)
PP (Hgmm, mean (SD))	49 (14)	45 (9)	48 (11)	54 (15)	48 (12)
Age (years, mean (SD))	49 (10)	38 (5)	31 (0)	61 (3)	38 (11)
Gender (male (%))	809 (51)	840 (45)	2531 (48)	444 (41)	4624 (47)
BMI (mean (SD))	27 (4)	25 (5)	25 (4)	27 (4)	25 (4)

WG: whole-genome, QC: quality control, BP: blood pressure, SBP: systolic blood pressure, DBP: diastolic blood pressure, MAP: mean arterial pressure, PP: pulse pressure, BMI: body-mass index, SD: 1 standard deviation.

The ICBP-GWAS represents a union of numerous prior blood pressure GWAS consortia to create a discovery meta-analysis of over 200 000 individuals of European ancestry. NFBC1966 is part of the ICBP-GWAS; however, this overlap does not represent a significant risk for bias, due to the small relative contribution of NFBC1966 to the ICBP-GWAS results.

### Genotyping and Imputation

All Finnish cohorts were genotyped using Illumina arrays (Illumina Inc. San Diego, CA, USA): Illumina Infinium HD Human610-Quad BeadChip for H2000, Illumina HumanCNV370-Duo BeadChip for NFBC1966, and Illumina Human670K custom BeadChip for YFS and HBCS. For SNPs to be successfully genotyped, a per individual and per marker success rate minimum of 95% was defined as default. 36 out of 41 candidate SNPs were successfully genotyped in all cohorts. For SNPs with no directly genotyped data available, we imputed genotypes with MACH [36] using HapMap CEU from Phase II as the reference panel (further referred to as HM2 imputed data). If a SNP was not present in HM2 imputed data, we used genotypes imputed with IMPUTEv2 using the 1000 Genomes pilot data CEU panel (August 2009 haplotypes) combined with HapMap Phase 3 (Public Release #2) haplotypes as the reference panel [37], extended with Finnish specific HapMap Phase 3 haplotypes [38] (further referred to as 1000G+HM3 imputed data). All missing genotypes were imputed, so the number of individuals included in the analyses for each SNP is the same and equals the final number (Table 4).

### IA Loci Association Analysis with Blood Pressure and Meta-Analysis of Results

Candidate loci were selected based on IA GWAS results [13]. Loci associated with IA with PPA≥0.1 were included (Table 2). PPA was calculated as described by Yasuno et al [12]. Briefly, a uniform prior probability of association of 1/10 000 was assumed for all SNPs and used to provide a probabilistic measure of evidence. We tested 41 SNPs from 19 independent loci. We defined SBP, DBP, MAP, and PP as quantitative outcome variables. MAP was counted as the average of SBP and DBP ((SBP+DBP)/2) and PP as the difference of the two (SBP-DBP). We tested all 19 loci in the discovery cohort (H2000), and those showing suggestive association (uncorrected p<0.1) with any outcome variable were tested in the replication cohorts (YFS, NFBC1966, and HBCS). Association analyses with an additive genetic model were performed with ProbABEL [39] for HM2 imputed data, and with SNPTESTv2 [40], [41] for 1000G+HM3 imputed data. The analyses were adjusted for age and gender (ROBUST model), or for age, gender, smoking habits, alcohol consumption, and BMI (ADVANCED model). Additionally, in the metabolic syndrome case-control subset of the H2000 cohort we corrected for case-control status in both models. Population stratification was corrected for by calculating principal components from genome-wide SNP data and including significant principal components in the association models as covariates. Association results were combined in a fixed effect meta-analysis with MetABEL [39] for HM2 imputed data, and with METAv1.2 [42] for 1000G+HM3 imputed data. The best result at 5q23.2 in *PRDM6* was tested for association in the ICBP-GWAS [19] cohort of 200 000 individuals of European descent. In the ICBP-GWAS association with SBP was tested by linear regression assuming an additive model and correcting for age, age-squared and BMI. To test the per-allele effect size of risk alleles on blood pressure, we calculated the mean blood pressure for the three genotypic states for the three 5q23.2 SNPs using Plink v1.07 [43]. Results were plotted using the Microsoft Excel charts function.

To further investigate the strongest associated locus, we analyzed all 1000 Genomes variants, with minor allele frequency greater than 1%, in and around *PRDM6*. We took uncertainty of imputation into account by using the maximum likelihood estimates of the reference allele counts as genotypes (these estimates may be fractional and range from 0 to 2). Fine mapping of the 5q23.2 region was performed with 1000G+HM3 imputed data. Results were plotted with LocusZoom [44].

## Supporting Information

Figure S1 Suggestive association with IA at 5q23.2 in GWAS (Yasuno et al 2010) [12]. The association to IA is strongest in the Finnish population, however, tendency is observable in other populations as well. FI = Finnish, NL = Dutch, DE = German, AN = mixed European cohort collected from Germany, Great Britain, Hungary, The Netherlands, Switzerland and Spain. JP2 = Japanese cohort.
(PDF)

Figure S2 Age distributions in the Finnish cohorts. In the NFBC1966 all were of the same age, since data utilized here were collected when the participants of the birth cohort were 31 years old. HBCS participants were older than the rest. (X-axis density = number of cohort participants).
(PDF)

Figure S3 Comparing causality and pleiotropy as possible explanations of the overlapping association between IA and SBP.
(PDF)

Table S1 Association results in the discovery cohort (H2000) of representative SNPs from the 19 regions tested. Association with systolic blood pressure (SBP), diastolic blood pressure (DBP), mean arterial pressure (MAP) and pulse pressure (PP) were tested with the ROBUST model (age and gender as covariates).
(DOC)

Table S2 2q33.1 and 5q23.2 loci cohort-wise ADVANCED model effect estimates and meta-analysis results with diastolic blood pressure (DBP) and mean arterial pressure (MAP).
(DOC)

Text S1 Full list of The International Consortium for Blood Pressure Genome-Wide Association Studies (ICBP-GWAS) co-authors, with affiliations.
(DOC)
