# Supplementary figures and images for: Intracranial Aneurysm Risk Locus 5q23.2 Is Associated with Elevated Systolic Blood Pressure
Source: PLoS Genet. 2012 Mar 15;8(3):e1002563. doi: 10.1371/journal.pgen.1002563 (PMC3305343; doi:10.1371/journal.pgen.1002563)

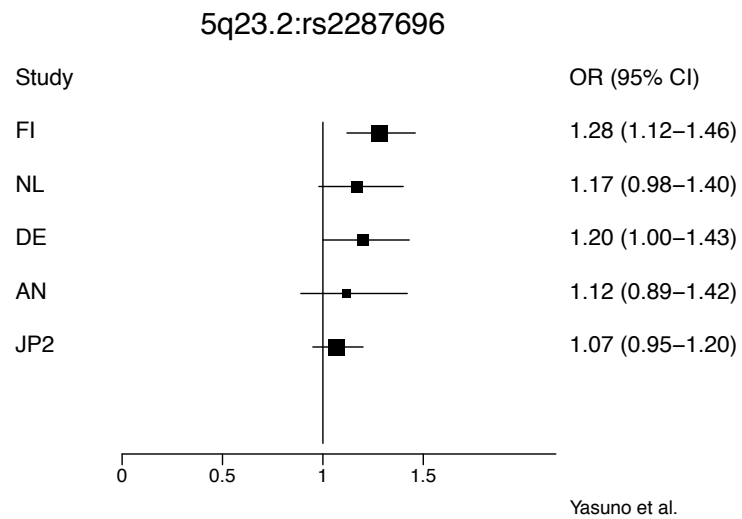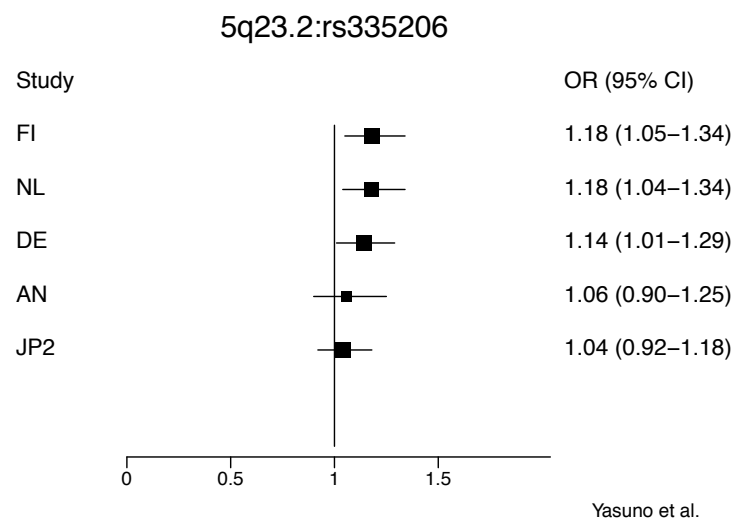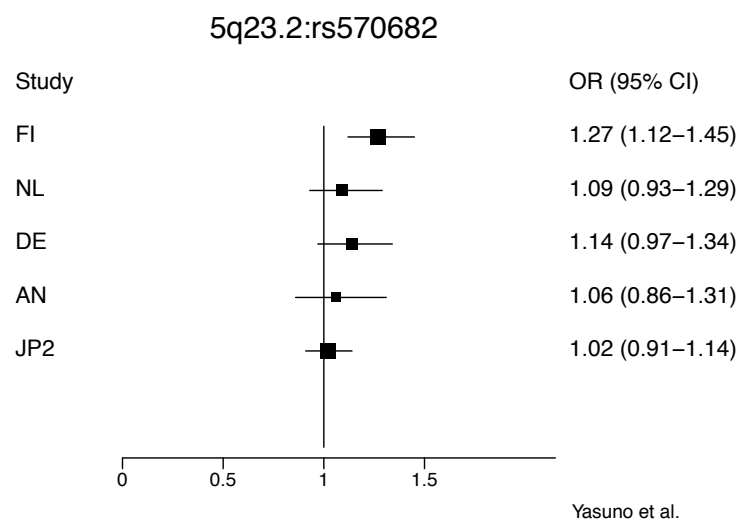

Supplement: Figure S1 — Suggestive association with IA at 5q23.2 in GWAS (Yasuno et al 2010) [12]. The association to IA is strongest in the Finnish population, however, tendency is observable in other populations as well. FI = Finnish, NL = Dutch, DE = German, AN = mixed European cohort collected from Germany, Great Britain, Hungary, The Netherlands, Switzerland and Spain. JP2 = Japanese cohort. (PDF) [file pgen.1002563.s001.pdf]

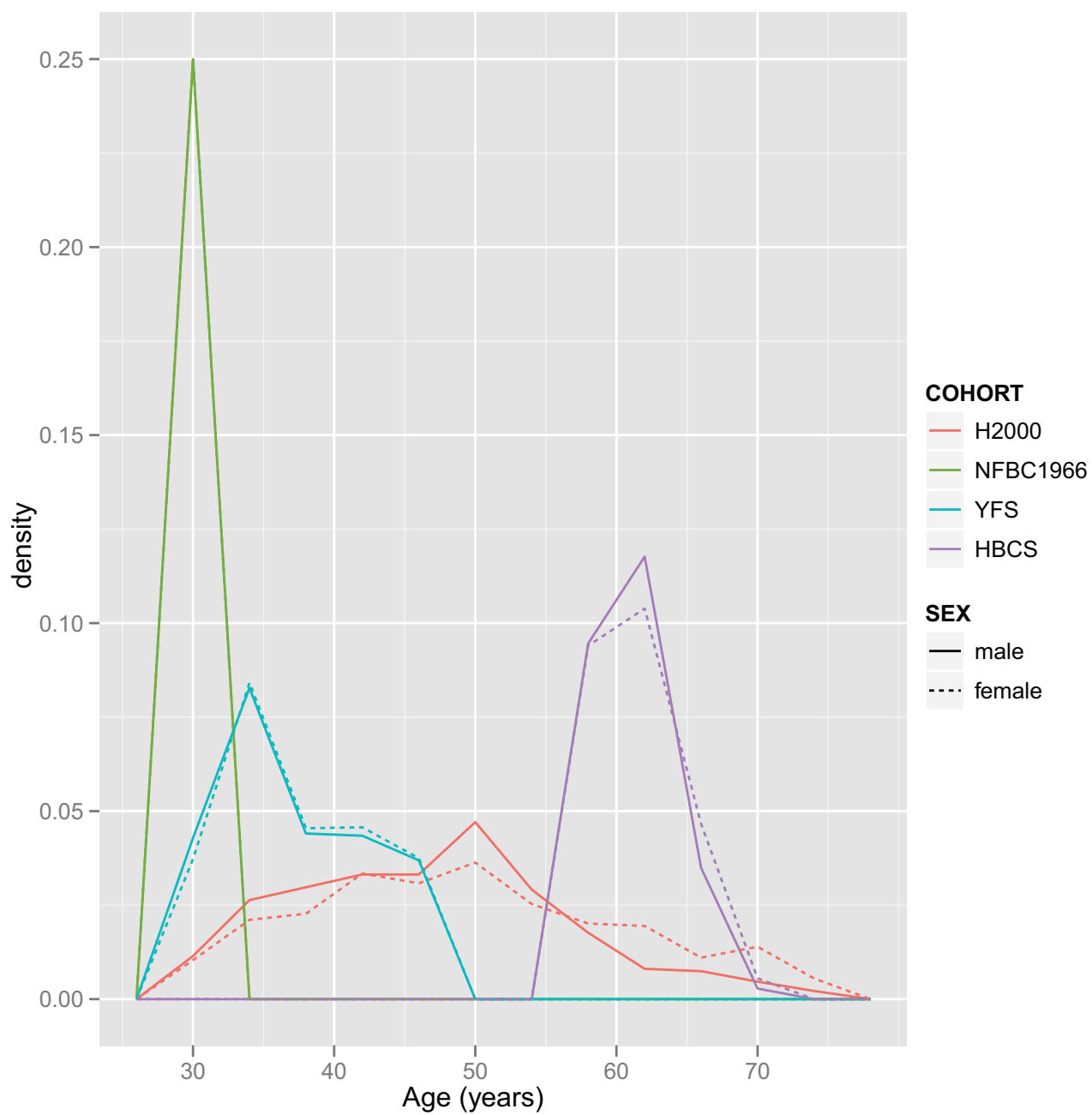

Supplement: Figure S2 — Age distributions in the Finnish cohorts. In the NFBC1966 all were of the same age, since data utilized here were collected when the participants of the birth cohort were 31 years old. HBCS participants were older than the rest. (X-axis density = number of cohort participants). (PDF) [file pgen.1002563.s002.pdf]

## Causality

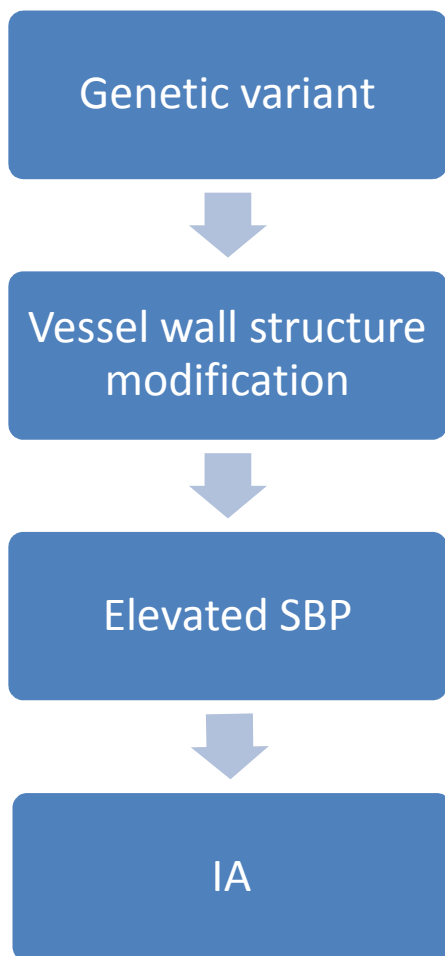

## Pleiotropy

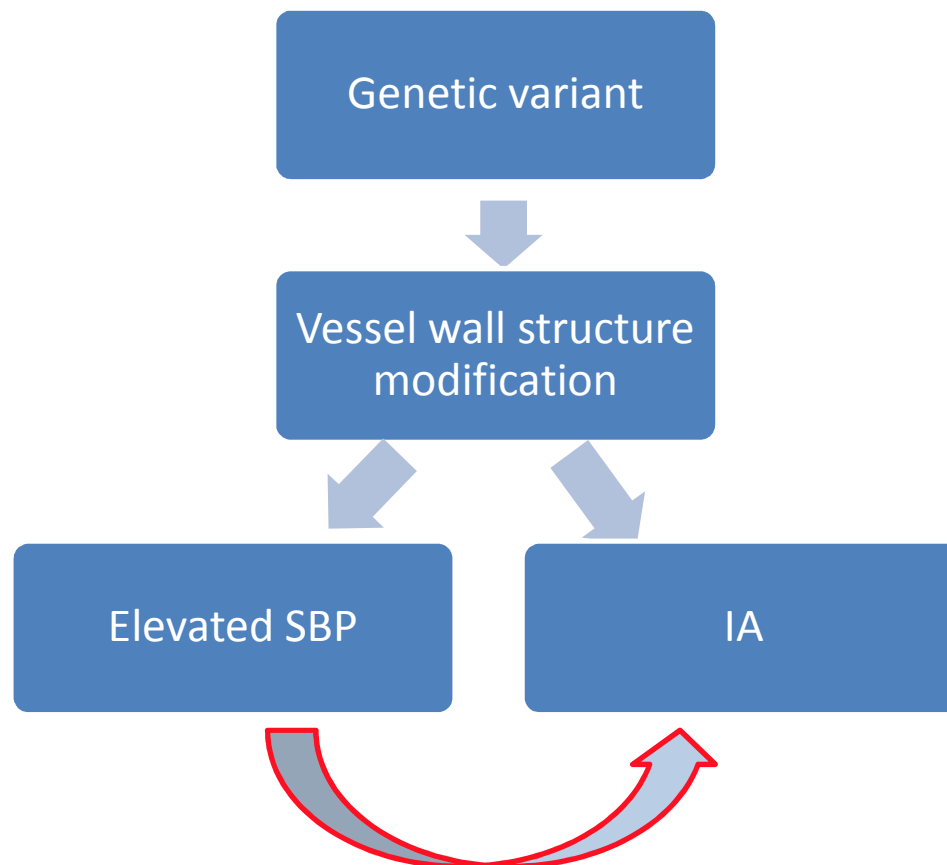

Supplement: Figure S3 — Comparing causality and pleiotropy as possible explanations of the overlapping association between IA and SBP. (PDF) [file pgen.1002563.s003.pdf]
