# Supplementary material for: Intracranial Aneurysm Risk Locus 5q23.2 Is Associated with Elevated Systolic Blood Pressure
Source: PLoS Genet. 2012 Mar 15;8(3):e1002563. doi: 10.1371/journal.pgen.1002563 (PMC3305343; doi:10.1371/journal.pgen.1002563)
Supplement: Table S1 — Association results in the discovery cohort (H2000) of representative SNPs from the 19 regions tested. Association with systolic blood pressure (SBP), diastolic blood pressure (DBP), mean arterial pressure (MAP) and pulse pressure (PP) were tested with the ROBUST model (age and gender as covariates). (DOC) [file pgen.1002563.s004.doc]

|  |  |  |  |  |  |  |  |  |  |  |
| --- | --- | --- | --- | --- | --- | --- | --- | --- | --- | --- |
| *Representative SNPs of loci with PPA>0.5 with IA (Yasuno et al 2010)* | | | | | | |  |  |  |  |
|  |  |  | SBP | | DBP | | MAP | PP |  |  |
| Locus | SNP | Effect Allele^#^ | beta (SE) | p | beta (SE) | p | beta (SE) | p |  |  |
| 8q12.1 | rs9298506* | G | 0.90 (0.71) | 2.06E-01 | 0.01 (0.44) | 9.82E-01 | 0.49 (0.52) | 1.18E-01 |  |  |
| 9p21.3 | rs1333040 | C | 0.25 (0.61) | 6.87E-01 | 0.39 (0.38) | 3.03E-01 | 0.28 (0.45) | 7.87E-01 |  |  |
| 10q24.32 | rs12413409 | A | -0.58 (1.09) | 5.92E-01 | -0.02 (0.67) | 9.78E-01 | -0.27 (0.79) | 8.42E-01 |  |  |
| 13q13.1 | rs9315204 | T | -0.61 (0.67) | 3.60E-01 | -0.37 (0.41) | 3.68E-01 | -0.51 (0.49) | 5.14E-01 |  |  |
| 18q11.2 | rs11661542 | C | -0.28 (0.61) | 6.48E-01 | -0.10 (0.38) | 7.86E-01 | -0.23 (0.45) | 8.75E-01 |  |  |
|  |  |  |  |  |  |  |  |  |  |  |
|  |  |  |  |  |  |  |  |  |  |  |
| *Representative SNPs of loci with 0.1≤PPA<0.5 with IA (Yasuno et al 2011/2)* | | | | | |  |  |  |  |  |
|  |  |  | SBP | | DBP | | MAP | PP |  |  |
| Locus | SNP | Effect Allele^#^ | beta (SE) | p | beta (SE) | p | beta (SE) | p |  |  |
| 1p36.31 | rs1876848 | A | -0.80 (0.73) | 2.76E-01 | -0.62 (0.45) | 1.67E-01 | -0.68 (0.54) | 6.18E-01 |  |  |
| 1p22.2 | rs1725390 | C | -0.66 (0.61) | 2.75E-01 | -0.10 (0.37) | 7.93E-01 | -0.39 (0.44) | 2.11E-01 |  |  |
| 1q21.3 | rs905938 | C | 0.06 (0.72) | 9.39E-01 | 0.31 (0.44) | 4.79E-01 | 0.18 (0.52) | 9.70E-01 |  |  |
| **2q33.1** | rs787994 | T | -0.69 (0.65) | 2.88E-01 | -0.66 (0.40) | 9.90E-02 | -0.66 (0.47) | 9.63E-01 |  |  |
| **4q31.23** | rs6841581* | A | -0.55 (0.93) | 5.51E-01 | 0.18 (0.57) | 7.54E-01 | -0.23 (0.68) | 2.19E-01 |  |  |
| **5q23.2** | rs2287696 | A | -1.83 (0.71) | 1.00E-02 | -0.77 (0.44) | 7.90E-02 | -1.27 (0.52) | 1.25E-01 |  |  |
| 8p23.2 | rs2045637 | A | 0.44 (0.90) | 6.28E-01 | 0.22 (0.56) | 6.95E-01 | 0.25 (0.66) | 5.73E-01 |  |  |
| 8q24.23 | rs6577930** | T | 0.03 (0.05) | 4.96E-01 | -0.36 (0.05) | 4.77E-01 | -0.02 (0.04) | 8.57E-01 |  |  |
| 11q22.2 | rs2124216 | A | 0.30 (0.72) | 6.74E-01 | 0.06 (0.44) | 8.92E-01 | 0.19 (0.53) | 7.12E-01 |  |  |
| 12p13.31 | rs728342 | T | 0.07 (0.67) | 9.14E-01 | 0.37 (0.41) | 3.73E-01 | 0.23 (0.49) | 6.14E-01 |  |  |
| 12q22 | rs6538595 | A | 0.44 (0.67) | 5.16E-01 | -0.04 (0.42) | 9.24E-01 | 0.15 (0.49) | 4.12E-01 |  |  |
| **19q13.12** | rs1688005 | T | 0.59 (0.65) | 3.62E-01 | 0.91 (0.40) | 2.30E-02 | 0.76 (0.48) | 6.33E-01 |  |  |
| 20p12.1 | rs1132274** | T | 0.02 (0.05) | 7.06E-01 | 0.06 (0.05) | 2.30E-01 | 0.05 (0.05) | 6.50E-01 |  |  |
| 22q12.1 | rs133885 | A | -0.32 (0.60) | 5.91E-01 | 0.02 (0.37) | 9.53E-01 | -0.16 (0.44) | 3.14E-01 |  |  |

SNPs are directly genotyped unless otherwise marked (* HM2 imputed SNP, ** 1000G+HM3 imputed SNP). Yasuno et al (2011/2) at 8q24.23 followed-up with rs1554349 instead of the lead SNP, rs6577930.

**In bold**: loci showing suggestive association (p<0.1) in the discovery cohort. At 4q31.23 the association was seen with DBP at rs1688005 (p=0.02).

^#^ Beta(SE) are counted for the Effect Alleles. SE: standard error.
