# Supplementary material for: Intracranial Aneurysm Risk Locus 5q23.2 Is Associated with Elevated Systolic Blood Pressure
Source: PLoS Genet. 2012 Mar 15;8(3):e1002563. doi: 10.1371/journal.pgen.1002563 (PMC3305343; doi:10.1371/journal.pgen.1002563)
Supplement: Table S2 — 2q33.1 and 5q23.2 loci cohort-wise ADVANCED model effect estimates and meta-analysis results with diastolic blood pressure (DBP) and mean arterial pressure (MAP). (DOC) [file pgen.1002563.s005.doc]

|  |  |  |  | Discovery - beta (SE) | Replication - beta (SE) | | | Meta DBP * | |
| --- | --- | --- | --- | --- | --- | --- | --- | --- | --- |
| Locus | SNP | IA Risk Allele | MAF | H2000 | YFS | NFBC1966 | HBCS | p | beta (SE) |
| 2q33.1 | rs1429412 | G | 0.5 | 0.51 (0.39) | 0.10 (0.34) | -0.06 (0.21) | 0.33 (0.43) | 4.78E-01 | 0.11 (0.15) |
| 2q33.1 | rs12472355 | A | 0.5 | 0.51 (0.39) | 0.13 (0.34) | 0.00 (0.21) | 0.31 (0.43) | 3.41E-01 | 0.15 (0.15) |
| 2q33.1 | rs787997 | A | 0.4 | 0.50 (0.39) | 0.10 (0.34) | 0.03 (0.22) | 0.35 (0.43) | 3.11E-01 | 0.16 (0.15) |
| 2q33.1 | rs787994 | T | 0.4 | 0.57 (0.40) | 0.20 (0.34) | 0.08 (0.22) | 0.39 (0.43) | 1.55E-01 | 0.22 (0.15) |
| 5q23.2 | rs570682 | T | 0.2 | 0.78 (0.42) | 0.14 (0.38) | 0.32 (0.25) | 0.68 (0.50) | 2.03E-02 | 0.40 (0.17) |
| 5q23.2 | rs2287696 | A | 0.2 | 0.76 (0.43) | 0.06 (0.39) | 0.24 (0.25) | 0.86 (0.52) | 4.20E-02 | 0.36 (0.18) |
| 5q23.2 | rs335206 | C | 0.4 | 0.36 (0.37) | 0.08 (0.33) | 0.34 (0.21) | 0.61 (0.42) | 3.23E-02 | 0.32 (0.15) |
|  |  |  |  |  |  |  |  |  |  |
|  |  |  |  | Discovery - beta (SE) | Replication - beta (SE) | | | Meta MAP * | |
| Locus | SNP | IA Risk Allele | MAF | H2000 | YFS | NFBC1966 | HBCS | p | beta (SE) |
| 2q33.1 | rs1429412 | G | 0.5 | 0.59 (0.47) | -0.06 (0.35) | -0.11 (0.20) | 0.19 (0.59) | 9.96E-01 | 0.00 (0.15) |
| 2q33.1 | rs12472355 | A | 0.5 | 0.59 (0.46) | -0.03 (0.35) | -0.05 (0.20) | 0.14 (0.59) | 7.99E-01 | 0.04 (0.16) |
| 2q33.1 | rs787997 | A | 0.4 | 0.52 (0.47) | 0.02 (0.35) | -0.02 (0.20) | 0.30 (0.58) | 6.52E-01 | 0.07 (0.16) |
| 2q33.1 | rs787994 | T | 0.4 | 0.60 (0.48) | 0.14 (0.35) | 0.03 (0.20) | 0.32 (0.59) | 3.97E-01 | 0.13 (0.16) |
| 5q23.2 | rs570682 | T | 0.2 | 1.11 (0.51) | 0.69 (0.39) | 0.47 (0.23) | 0.57 (0.68) | 7.03E-04 | 0.60 (0.18) |
| 5q23.2 | rs2287696 | A | 0.2 | 1.22 (0.52) | 0.63 (0.41) | 0.43 (0.24) | 0.89 (0.71) | 1.01E-03 | 0.60 (0.18) |
| 5q23.2 | rs335206 | C | 0.4 | 0.68 (0.44) | 0.47 (0.34) | 0.53 (0.20) | 0.60 (0.58) | 4.09E-04 | 0.54 (0.15) |

Genomic positions are based on the human genome build 36. Alleles are reported on the forward strand of the reference genome. The effects are reported for the alleles increasing risk for IA in the Yasuno et al. studies (2011/1&2). Risk alleles are aligned according to the forward strand of the reference genome. Minor allele frequencies (MAF) are based on from the HapMap Phase II CEU population data.

* Meta DBP and Meta MAP: meta-analysis of discovery and replication cohorts p-values and beta for diastolic blood pressure (DBP) and mean arterial pressure (MAP) with the ADVANCED model. Association analyses were corrected for gender, age, BMI, smoking habits and alcohol consumption.

SE: standard error
