# Supplementary material for: Intracranial Aneurysm Risk Locus 5q23.2 Is Associated with Elevated Systolic Blood Pressure
Source: PLoS Genet. 2012 Mar 15;8(3):e1002563. doi: 10.1371/journal.pgen.1002563 (PMC3305343; doi:10.1371/journal.pgen.1002563)
Supplement: Text S1 — Full list of The International Consortium for Blood Pressure Genome-Wide Association Studies (ICBP-GWAS) co-authors, with affiliations. (DOC) [file pgen.1002563.s006.doc]

**Text S1**

Full list of ICBP-GWAS co-authors

Georg B. Ehret^1,2,3^, Patricia B. Munroe^4^, Kenneth M. Rice^5^, Murielle Bochud^2^, Andrew D. Johnson^6,7^, Daniel I. Chasman^8,9^, Albert V. Smith^10,11^, Martin D. Tobin^12^, Germaine C. Verwoert^13,14,15^, Shih-Jen Hwang^6,16,7^, Vasyl Pihur^1^, Peter Vollenweider^17^, Paul F. O'Reilly^18^, Najaf Amin^13^, Jennifer L Bragg-Gresham^19^, Alexander Teumer^20^, Nicole L. Glazer^21^, Lenore Launer^22^, Jing Hua Zhao^23^, Yurii Aulchenko^13^, Simon Heath^24^, Siim Sõber^25^, Afshin Parsa^26^, Jian'an Luan^23^, Pankaj Arora^27^, Abbas Dehghan^13,14,15^, Feng Zhang^28^, Gavin Lucas^29^, Andrew A. Hicks^30^, Anne U. Jackson^31^, John F Peden^32^, Toshiko Tanaka^33^, Sarah H. Wild^34^, Igor Rudan^35,36^, Wilmar Igl^37^, Yuri Milaneschi^33^, Alex N. Parker^38^, Cristiano Fava^39,40^, John C. Chambers^18,41^, Ervin R. Fox^42^, Meena Kumari^43^, Min Jin Go^44^, Pim van der Harst^45^, Wen Hong Linda Kao^46^, Marketa Sjögren^39^, D. G. Vinay^47^, Myriam Alexander^48^, Yasuharu Tabara^49^, Sue Shaw-Hawkins^4^, Peter H. Whincup^50^, Yongmei Liu^51^, Gang Shi^52^, Johanna Kuusisto^53^, Bamidele Tayo^54^, Mark Seielstad^55,56^, Xueling Sim^57^, Khanh-Dung Hoang Nguyen^1^, Giuseppe Matullo^59,60^, Ying Wu^61^, Tom R. Gaunt^62^, N. Charlotte Onland-Moret^63,64^, Matthew N. Cooper^65^, Carl G.P. Platou^66^, Elin Org^25^, Rebecca Hardy^67^, Santosh Dahgam^68^, Jutta Palmen^69^, Veronique Vitart^70^, Peter S. Braund^71,72^, Tatiana Kuznetsova^73^, Cuno S.P.M. Uiterwaal^63^, Adebowale Adeyemo^74^, Walter Palmas^75^, Harry Campbell^35^, Barbara Ludwig^76^, Maciej Tomaszewski^71,72^, Ioanna Tzoulaki^77,78^, Nicholette D. Palmer^79^, CARDIoGRAM consortium^80^, CKDGen Consortium^80^, KidneyGen Consortium^80^, EchoGen consortium^80^, CHARGE-HF consortium^80^, Thor Aspelund^10,11^, Melissa Garcia^22^, Yen-Pei C. Chang^26^, Jeffrey R. O'Connell^26^, Nanette I. Steinle^26^, Diederick E. Grobbee^63^, Dan E. Arking^1^, Sharon L. Kardia^81^, Alanna C. Morrison^82^, Dena Hernandez^83^, Samer Najjar^84,85^, Wendy L. McArdle^86^, David Hadley^50,87^, Morris J. Brown^88^, John M. Connell^89^, Aroon D. Hingorani^90^, Ian N.M. Day^62^, Debbie A. Lawlor^62^, John P. Beilby^91,92^, Robert W. Lawrence^65^, Robert Clarke^93^, Rory Collins^93^, Jemma C Hopewell^93^, Halit Ongen^32^, Albert W. Dreisbach^42^, Yali Li^94^, J H. Young^95^, Joshua C. Bis^21^, Mika Kähönen^96^, Jorma Viikari^97^, Linda S. Adair^98^, Nanette R. Lee^99^, Ming-Huei Chen^100^, Matthias Olden^101,102^, Cristian Pattaro^30^, Judith A. Hoffman Bolton^103^, Anna Köttgen^104,103^, Sven Bergmann^105,106^, Vincent Mooser^107^, Nish Chaturvedi^108^, Timothy M. Frayling^109^, Muhammad Islam^110^, Tazeen H. Jafar^110^, Jeanette Erdmann^111^, Smita R. Kulkarni^112^, Stefan R. Bornstein^76^, Jürgen Grässler^76^, Leif Groop^113,114^, Benjamin F. Voight^115^, Philip Howard^117^, Andrew Taylor^43^, Simonetta Guarrera^60^, Fulvio Ricceri^59,60^, Valur Emilsson^118^, Andrew Plump^118^, Inês Barroso^119,120^, Kay-Tee Khaw^48^, Alan B. Weder^121^, Steven C. Hunt^122^, Yan V. Sun^81^, Richard N. Bergman^123^, Francis S. Collins^124^, Lori L. Bonnycastle^124^, Laura J. Scott^31^, Heather M. Stringham^31^, Leena Peltonen^119,125,126,127^, Erkki Vartiainen^125^, Stefan-Martin Brand^128,129^, Jan A. Staessen^73^, Thomas J. Wang^6,130^, Paul R. Burton^12,72^, Maria Soler Artigas^12^, Yanbin Dong^131^, Harold Snieder^132,131^, Xiaoling Wang^131^, Haidong Zhu^131^, Kurt K. Lohman^133^, Megan E. Rudock^51^, Susan R Heckbert^134,135^, Nicholas L Smith^134,136,135^, Kerri L Wiggins^137^, Ayo Doumatey^74^, Daniel Shriner^74^, Gudrun Veldre^25,138^, Margus Viigimaa^139,140^, Sanjay Kinra^141^, Dorairajan Prabhakaran^142^, Vikal Tripathy^142^, Carl D. Langefeld^79^, Annika Rosengren^143^, Dag S. Thelle^144^, Anna Maria Corsi^145^, Andrew Singleton^83^, Terrence Forrester^146^, Gina Hilton^1^, Colin A. McKenzie^146^, Tunde Salako^147^, Naoharu Iwai^148^, Yoshikuni Kita^149^, Toshio Ogihara^150^, Takayoshi Ohkubo^149,151^, Tomonori Okamura^148^, Hirotsugu Ueshima^152^, Satoshi Umemura^153^, Susana Eyheramendy^154^, Thomas Meitinger^155,156^, H.-Erich Wichmann^157,158,159^, Yoon Shin Cho^44^, Hyung-Lae Kim^44^, Jong-Young Lee^44^, James Scott^160^, Joban S. Sehmi^160,41^, Weihua Zhang^18^, Bo Hedblad^39^, Peter Nilsson^39^, George Davey Smith^62^, Andrew Wong^67^, Narisu Narisu^124^, Alena Stančáková^53^, Leslie J. Raffel^161^, Jie Yao^161^, Sekar Kathiresan^162,27^, Chris O'Donnell^163,27,9^, Stephen M. Schwartz^134^, M. Arfan Ikram^13,15^, W. T. Longstreth Jr.^164^, Thomas H. Mosley^165^, Sudha Seshadri^166^, Nick R.G. Shrine^12^, Louise V. Wain^12^, Mario A. Morken^124^, Amy J. Swift^124^, Jaana Laitinen^167^, Inga Prokopenko^51,168^, Paavo Zitting^169^, Jackie A. Cooper^69^, Steve E. Humphries^69^, John Danesh^48^, Asif Rasheed^170^, Anuj Goel^32^, Anders Hamsten^171^, Hugh Watkins^32^, Stephan J.L. Bakker^172^, Wiek H. van Gilst^45^, Charles S. Janipalli^47^, K. Radha Mani^47^, Chittaranjan S. Yajnik^112^, Albert Hofman^13^, Francesco U.S. Mattace-Raso^13,14^, Ben A. Oostra^173^, Ayse Demirkan^13^, Aaron Isaacs^13^, Fernando Rivadeneira^13,14^, Edward G Lakatta^174^, Marco Orru^175,176^, Angelo Scuteri^174^, Mika Ala-Korpela^177,178,179^, Antti J Kangas^177^, Leo-Pekka Lyytikäinen^58^, Pasi Soininen^177,178^, Taru Tukiainen^180,181,177^, Peter Würtz^177,18,180^, Rick Twee-Hee Ong^56,57,182^, Marcus Dörr^183^, Heyo K. Kroemer^184^, Uwe Völker^20^, Henry Völzke^185^, Pilar Galan^186^, Serge Hercberg^186^, Mark Lathrop^24^, Diana Zelenika^24^, Panos Deloukas^119^, Massimo Mangino^28^, Tim D. Spector^28^, Guangju Zhai^28^, James F. Meschia^187^, Michael A. Nalls^83^, Pankaj Sharma^188^, Janos Terzic^189^, M. J. Kranthi Kumar^47^, Matthew Denniff^71^, Ewa Zukowska-Szczechowska^190^, Lynne E. Wagenknecht^79^, F. Gerald R. Fowkes^191^, Fadi J. Charchar^192^, Peter E.H. Schwarz^193^, Caroline Hayward^70^, Xiuqing Guo^161^, Charles Rotimi^74^, Michiel L. Bots^63^, Eva Brand^194^, Nilesh J. Samani^71,72^, Ozren Polasek^195^, Philippa J. Talmud^69^, Fredrik Nyberg^68,196^, Diana Kuh^67^, Maris Laan^25^, Kristian Hveem^66^, Lyle J. Palmer^197,198^, Yvonne T. van der Schouw^63^, Juan P. Casas^199^, Karen L. Mohlke^61^, Paolo Vineis^200,60^, Santhi K. Ganesh^202^, Tien Y. Wong^203,204^, E Shyong Tai^205,57,206^, Richard S. Cooper^54^, Markku Laakso^53^, Dabeeru C. Rao^207^, Tamara B. Harris^22^, Richard W. Morris^208^, Anna F. Dominiczak^209^, Mika Kivimaki^210^, Michael G. Marmot^210^, Tetsuro Miki^49^, Danish Saleheen^170,48^, Giriraj R. Chandak^47^, Josef Coresh^211^, Gerjan Navis^212^, Bok-Ghee Han^44^, Xiaofeng Zhu^94^, Jaspal S. Kooner^160,41^, Olle Melander^39^, Paul M Ridker^8,213,9^, Stefania Bandinelli^214^, Ulf B. Gyllensten^37^, Alan F. Wright^70^, James F. Wilson^34^, Luigi Ferrucci^33^, Martin Farrall^32^, Jaakko Tuomilehto^215,216,217,218^, Peter P. Pramstaller^30,219^, Roberto Elosua^29,220^, Nicole Soranzo^119,28^, Eric J.G. Sijbrands^13,14^, David Altshuler^221,115^, Ruth J.F. Loos^23^, Alan R. Shuldiner^26,222^, Christian Gieger^157^, Pierre Meneton^223^, Andre G. Uitterlinden^13,14,15^, Nicholas J. Wareham^23^, Vilmundur Gudnason^10,11^, Jerome I. Rotter^161^, Rainer Rettig^224^, Manuela Uda^175^, David P. Strachan^50^, Jacqueline C.M. Witteman^13,15^, Anna-Liisa Hartikainen^225^, Jacques S. Beckmann^105,226^, Eric Boerwinkle^227^, Ramachandran S. Vasan^6,228^, Michael Boehnke^31^, Martin G. Larson^6,229^, Bruce M. Psaty^21,135*^, Gonçalo R Abecasis^19*^, Aravinda Chakravarti^1*^, Paul Elliott^18,233*^, Cornelia M. van Duijn^13,234*^, Daniel Levy^6,16,7*^, Mark J. Caulfield^4*^, Toby Johnson^4*^

ICBP-GWAS co-authors’ affiliations

1. Center for Complex Disease Genomics, McKusick-Nathans Institute of Genetic Medicine, Johns Hopkins University School of Medicine, Baltimore, MD 21205, USA

2. Institute of Social and Preventive Medicine (IUMSP), Centre Hospitalier Universitaire Vaudois and University of Lausanne, Bugnon 17, 1005 Lausanne, Switzerland

3. Cardiology, Department of Specialties of Internal Medicine, Geneva University Hospital, Rue Gabrielle-Perret-Gentil 4, 1211 Geneva 14, Switzerland

4. Clinical Pharmacology and The Genome Centre, William Harvey Research Institute, Barts and The London School of Medicine and Dentistry, Queen Mary University of London, London EC1M 6BQ, UK

5. Department of Biostatistics, University of Washington, Seattle, WA, USA

6. Framingham Heart Study, Framingham, MA, USA

7. National Heart Lung, and Blood Institute, Bethesda, MD, USA

8. Division of Preventive Medicine, Brigham and Women's Hospital, 900 Commonwealth Avenue East, Boston MA 02215, USA

9. Harvard Medical School, Boston, MA, USA

10. Icelandic Heart Association, Kopavogur, Iceland

11. University of Iceland, Reykajvik, Iceland

12. Department of Health Sciences, University of Leicester, University Rd, Leicester LE1 7RH, UK

13. Department of Epidemiology, Erasmus Medical Center, PO Box 2040, 3000 CA, Rotterdam, The Netherlands

14. Department of Internal Medicine, Erasmus Medical Center, Rotterdam, The Netherlands

15. Netherlands Consortium for Healthy Aging (NCHA), Netherland Genome Initiative (NGI), The Netherlands

16. Center for Population Studies, National Heart Lung, and Blood Institute, Bethesda, MD, USA

17. Department of Internal Medicine, Centre Hospitalier Universitaire Vaudois, 1011 Lausanne, Switzerland

18. Department of Epidemiology and Biostatistics, School of Public Health, Imperial College London, Norfolk Place, London W2 1PG, UK

19. Center for Statistical Genetics, Department of Biostatistics, University of Michigan School of Public Health, Ann Arbor, MI 48103, USA

20. Interfaculty Institute for Genetics and Functional Genomics, Ernst-Moritz-Arndt-University Greifswald, 17487 Greifswald, Germany

21. Cardiovascular Health Research Unit, Departments of Medicine, Epidemiology and Health Services, University of Washington, Seattle, WA, USA

22. Laboratory of Epidemiology, Demography, Biometry, National Institute on Aging, National Institutes of Health, Bethesda, Maryland 20892, USA

23. MRC Epidemiology Unit, Institute of Metabolic Science, Cambridge CB2 0QQ, UK

24. Centre National de Génotypage, Commissariat à L'Energie Atomique, Institut de Génomique, Evry, France

25. Institute of Molecular and Cell Biology, University of Tartu, Riia 23, Tartu 51010, Estonia

26. University of Maryland School of Medicine, Baltimore, MD, USA, 21201, USA

27. Center for Human Genetic Research, Cardiovascular Research Center, Massachusetts General Hospital, Boston, Massachusetts, 02114, USA

28. Department of Twin Research & Genetic Epidemiology, King's College London, UK

29. Cardiovascular Epidemiology and Genetics, Institut Municipal d'Investigacio Medica, Barcelona Biomedical Research Park, 88 Doctor Aiguader, 08003 Barcelona, Spain

30. Institute of Genetic Medicine, European Academy Bozen/Bolzano (EURAC), Viale Druso 1, 39100 Bolzano, Italy - Affiliated Institute of the University of Lübeck, Germany

31. Department of Biostatistics, Center for Statistical Genetics, University of Michigan, Ann Arbor, Michigan, 48109, USA

32. Department of Cardiovascular Medicine, The Wellcome Trust Centre for Human Genetics, University of Oxford, Oxford, OX3 7BN, UK

33. Clinical Research Branch, National Institute on Aging, Baltimore MD 21250, USA

34. Centre for Population Health Sciences, University of Edinburgh, EH89AG, UK

35. Centre for Population Health Sciences and Institute of Genetics and Molecular Medicine, College of Medicine and Vet Medicine, University of Edinburgh, EH8 9AG, UK

36. Croatian Centre for Global Health, University of Split, Croatia

37. Department of Genetics and Pathology, Rudbeck Laboratory, Uppsala University, SE-751 85 Uppsala, Sweden

38. Amgen, 1 Kendall Square, Building 100, Cambridge, MA 02139, USA

39. Department of Clinical Sciences, Lund University, Malmö, Sweden

40. Department of Medicine, University of Verona, Italy

41. Ealing Hospital, London, UB1 3HJ, UK

42. Department of Medicine, University of Mississippi Medical Center, USA

43. Genetic Epidemiology Group, Epidemiology and Public Health, UCL, London, WC1E 6BT, UK

44. Center for Genome Science, National Institute of Health, Seoul, Korea

45. Department of Cardiology, University Medical Center Groningen, University of Groningen, The Netherlands

46. Departments of Epidemiology and Medicine, Johns Hopkins University, Baltimore MD, USA

47. Centre for Cellular and Molecular Biology (CCMB), Council of Scientific and Industrial Research (CSIR), Uppal Road, Hyderabad 500 007, India

48. Department of Public Health and Primary Care, University of Cambridge, CB1 8RN, UK

49. Department of Basic Medical Research and Education, and Department of Geriatric Medicine, Ehime University Graduate School of Medicine, Toon, 791-0295, Japan

50. Division of Community Health Sciences, St George's University of London, London, SW17 0RE, UK

51. Epidemiology & Prevention, Division of Public Health Sciences, Wake Forest University School of Medicine, Winston-Salem, NC 27157, USA

52. Division of Biostatistics and Department of Genetics, School of Medicine, Washington University in St. Louis, Saint Louis, Missouri 63110, USA

53. Department of Medicine, University of Eastern Finland and Kuopio University Hospital, 70210 Kuopio, Finland

54. Department of Preventive Medicine and Epidemiology, Loyola University Medical School, Maywood, IL, USA

55. Department of Laboratory Medicine & Institute of Human Genetics, University of California San Francisco, 513 Parnassus Ave. San Francisco CA 94143, USA

56. Genome Institute of Singapore, Agency for Science, Technology and Research, Singapore, 138672, Singapore

57. Centre for Molecular Epidemiology, Yong Loo Lin School of Medicine, National University of Singapore, Singapore, 117597, Singapore

58. Department of Clinical Chemistry, University of Tampere and Tampere University Hospital, Tampere, 33521, Finland

59. Department of Genetics, Biology and Biochemistry, University of Torino, Via Santena 19, 10126, Torino, Italy

60. Human Genetics Foundation (HUGEF), Via Nizza 52, 10126, Torino, Italy

61. Department of Genetics, University of North Carolina, Chapel Hill, NC, 27599, USA

62. MRC Centre for Causal Analyses in Translational Epidemiology, School of Social & Community Medicine, University of Bristol, Bristol BS8 2BN, UK

63. Julius Center for Health Sciences and Primary Care, University Medical Center Utrecht, Heidelberglaan 100, 3508 GA Utrecht, The Netherlands

64. Complex Genetics Section, Department of Medical Genetics - DBG, University Medical Center Utrecht, 3508 GA Utrecht, The Netherlands

65. Centre for Genetic Epidemiology and Biostatistics, University of Western Australia, Crawley, WA, Australia

66. HUNT Research Centre, Department of Public Health and General Practice, Norwegian University of Science and Technology, 7600 Levanger, Norway

67. MRC Unit for Lifelong Health & Ageing, London, WC1B 5JU, UK

68. Occupational and Environmental Medicine, Department of Public Health and Community Medicine, Institute of Medicine, Sahlgrenska Academy, University of Gothenburg, 40530 Gothenburg, Sweden

69. Centre for Cardiovascular Genetics, University College London, London WC1E 6JF, UK

70. MRC Human Genetics Unit and Institute of Genetics and Molecular Medicine, Edinburgh, EH2, UK

71. Department of Cardiovascular Sciences, University of Leicester, Glenfield Hospital, Leicester, LE3 9QP, UK

72. Leicester NIHR Biomedical Research Unit in Cardiovascular Disease, Glenfield Hospital, Leicester, LE3 9QP, UK

73. Studies Coordinating Centre, Division of Hypertension and Cardiac Rehabilitation, Department of Cardiovascular Diseases, University of Leuven, Campus Sint Rafaël, Kapucijnenvoer 35, Block D, Box 7001, 3000 Leuven, Belgium

74. Center for Research on Genomics and Global Health, National Human Genome Research Institute, Bethesda, MD 20892, USA

75. Columbia University, NY, USA

76. Department of Medicine III, Medical Faculty Carl Gustav Carus at the Technical University of Dresden, 01307 Dresden, Germany

77. Epidemiology and Biostatistics, School of Public Health, Imperial College, London, W2 1PG, UK

78. Clinical and Molecular Epidemiology Unit, Department of Hygiene and Epidemiology, University of Ioannina School of Medicine, Ioannina, Greece

79. Wake Forest University Health Sciences, Winston-Salem, NC 27157, USA

80. CHARGE-HF consortium

81. Department of Epidemiology, School of Public Health, University of Michigan, Ann Arbor, MI 48109, USA

82. Division of Epidemiology, Human Genetics and Environmental Sciences, School of Public Health, University of Texas at Houston Health Science Center, 12 Herman Pressler, Suite 453E, Houston, TX 77030, USA

83. Laboratory of Neurogenetics, National Institute on Aging, Bethesda, MD 20892, USA

84. Laboratory of Cardiovascular Science, Intramural Research Program, National Institute on Aging, NIH, Baltimore, Maryland, USA

85. Washington Hospital Center, Division of Cardiology, Washington DC, USA

86. ALSPAC Laboratory, University of Bristol, Bristol, BS8 2BN, UK

87. Pediatric Epidemiology Center, University of South Florida, Tampa, FL, USA

88. Clinical Pharmacology Unit, University of Cambridge, Addenbrookes Hospital, Hills Road, Cambridge CB2 2QQ, UK

89. University of Dundee, Ninewells Hospital &Medical School, Dundee, DD1 9SY, UK

90. Genetic Epidemiology Group, Department of Epidemiology and Public Health, UCL, London WC1E 6BT, UK

91. Pathology and Laboratory Medicine, University of Western Australia, Crawley, WA, Australia

92. Molecular Genetics, PathWest Laboratory Medicine, Nedlands, WA, Australia

93. Clinical Trial Service Unit and Epidemiological Studies Unit, University of Oxford, Oxford, OX3 7LF, UK

94. Department of Epidemiology and Biostatistics, Case Western Reserve University, 2103 Cornell Road, Cleveland, OH 44106, USA

95. Department of Medicine, Johns Hopkins University, Baltimore, USA

96. Department of Clinical Physiology, University of Tampere and Tampere University Hospital, Tampere, 33521, Finland

97. Department of Medicine, University of Turku and Turku University Hospital, Turku, 20521, Finland

98. Department of Nutrition, University of North Carolina, Chapel Hill, NC, 27599, USA

99. Office of Population Studies Foundation, University of San Carlos, Talamban, Cebu City 6000, Philippines

100. Department of Neurology and Framingham Heart Study, Boston University School of Medicine, Boston, MA, 02118, USA

101. Department of Internal Medicine II, University Medical Center Regensburg, 93053 Regensburg, Germany

102. Department of Epidemiology and Preventive Medicine, University Medical Center Regensburg, 93053 Regensburg, Germany

103. Department of Epidemiology, Johns Hopkins University, Baltimore MD, USA

104. Renal Division, University Hospital Freiburg, Germany

105. Département de Génétique Médicale, Université de Lausanne, 1015 Lausanne, Switzerland

106. Swiss Institute of Bioinformatics, 1015 Lausanne, Switzerland

107. Division of Genetics, GlaxoSmithKline, Philadelphia, Pennsylvania 19101, USA

108. International Centre for Circulatory Health, National Heart & Lung Institute, Imperial College, London, UK

109. Genetics of Complex Traits, Peninsula Medical School, University of Exeter, UK

110. Department of Community Health Sciences & Department of Medicine, Aga Khan University, Karachi, Pakistan

111. Medizinische Klinik II, Universität zu Lübeck, Lübeck, Germany

112. Diabetes Unit, KEM Hospital and Research Centre, Rasta Peth, Pune-411011, Maharashtra, India

113. Department of Clinical Sciences, Diabetes and Endocrinology Research Unit, University Hospital, Malmö, Sweden

114. Lund University, Malmö 20502, Sweden

115. Program in Medical and Population Genetics, Broad Institute of Harvard and MIT, Cambridge, Massachusetts, 02139, USA

117. William Harvey Research Institute, Barts and The London School of Medicine and Dentistry, Queen Mary University of London, London EC1M 6BQ, UK

118. Merck Research Laboratory, 126 East Lincoln Avenue, Rahway, NJ 07065, USA

119. Wellcome Trust Sanger Institute, Hinxton, CB10 1SA, UK

120. University of Cambridge Metabolic Research Labs, Institute of Metabolic Science Addenbrooke's Hospital, CB2 OQQ, Cambridge, UK

121. Division of Cardiovascular Medicine, Department of Internal Medicine, University of Michigan Medical School, Ann Arbor, MI, USA

122. Cardiovascular Genetics, University of Utah School of Medicine, Salt Lake City, UT, USA

123. Department of Physiology and Biophysics, Keck School of Medicine, University of Southern California, Los Angeles, California 90033, USA

124. National Human Genome Research Institute, National Institutes of Health, Bethesda, Maryland 20892,USA

125. National Institute for Health and Welfare, 00271 Helsinki, Finland

126. FIMM, Institute for Molecular Medicine, Finland, Biomedicum, P.O. Box 104, 00251 Helsinki, Finland

127. Broad Institute, Cambridge, Massachusetts 02142, USA

128. Leibniz-Institute for Arteriosclerosis Research, Department of Molecular Genetics of Cardiovascular Disease, University of Münster, Münster, Germany

129. Medical Faculty of the Westfalian Wilhelms University Muenster, Department of Molecular Genetics of Cardiovascular Disease, University of Muenster, Muenster, Germany

130. Division of Cardiology, Massachusetts General Hospital, Boston, MA, USA

131. Georgia Prevention Institute, Department of Pediatrics, Medical College of Georgia, Augusta, GA, USA

132. Unit of Genetic Epidemiology and Bioinformatics, Department of Epidemiology, University Medical Center Groningen, University of Groningen, Groningen, The Netherlands

133. Department of Biostatical Sciences, Division of Public Health Sciences, Wake Forest University School of Medicine, Winston-Salem, NC 27157, USA

134. Department of Epidemiology, University of Washington, Seattle, WA, 98195, USA

135. Group Health Research Institute, Group Health Cooperative, Seattle, WA, USA

136. Seattle Epidemiologic Research and Information Center, Veterans Health Administration Office of Research & Development, Seattle, WA 98108, USA

137. Department of Medicine, University of Washington, 98195, USA

138. Department of Cardiology, University of Tartu, L. Puusepa 8, 51014 Tartu, Estonia

139. Tallinn University of Technology, Institute of Biomedical Engineering, Ehitajate tee 5, 19086 Tallinn, Estonia

140. Centre of Cardiology, North Estonia Medical Centre, Sütiste tee 19, 13419 Tallinn, Estonia

141. Division of Non-communicable disease Epidemiology, The London School of Hygiene and Tropical Medicine London, Keppel Street, London WC1E 7HT, UK

142. South Asia Network for Chronic Disease, Public Health Foundation of India, C-1/52, SDA, New Delhi 100016, India

143. Department of Emergency and Cardiovascular Medicine, Institute of Medicine, Sahlgrenska Academy, University of Gothenburg, 41685 Gothenburg, Sweden

144. Department of Biostatistics, Institute of Basic Medical Sciences, University of Oslo, 0317 Oslo, Norway

145. Tuscany Regional Health Agency, Florence, Italy

146. Tropical Medicine Research Institute, University of the West Indies, Mona, Kingston, Jamaica

147. University of Ibadan, Ibadan, Nigeria

148. Department of Genomic Medicine, and Department of Preventive Cardiology, National Cerebral and Cardiovascular Research Center, Suita, 565-8565, Japan

149. Department of Health Science, Shiga University of Medical Science, Otsu, 520-2192, Japan

150. Department of Geriatric Medicine, Osaka University Graduate School of Medicine, Suita, 565-0871, Japan

151. Tohoku University Graduate School of Pharmaceutical Sciences and Medicine, Sendai, 980-8578, Japan

152. Lifestyle-related Disease Prevention Center, Shiga University of Medical Science, Otsu, 520-2192, Japan

153. Department of Medical Science and Cardiorenal Medicine, Yokohama City University School of Medicine, Yokohama, 236-0004, Japan

154. Department of Statistics, Pontificia Universidad Catolica de Chile, Vicuña Mackena 4860, Santiago, Chile

155. Institute of Human Genetics, Helmholtz Zentrum Munich, German Research Centre for Environmental Health, 85764 Neuherberg, Germany

156. Institute of Human Genetics, Klinikum rechts der Isar, Technical University of Munich, 81675 Munich, Germany

157. Institute of Epidemiology, Helmholtz Zentrum Munich, German Research Centre for Environmental Health, 85764 Neuherberg, Germany

158. Chair of Epidemiology, Institute of Medical Informatics, Biometry and Epidemiology, Ludwig-Maximilians-Universität, 81377 Munich, Germany

159. Klinikum Grosshadern, 81377 Munich, Germany

160. National Heart and Lung Institute, Imperial College London, London, UK, W12 0HS, UK

161. Medical Genetics Institute, Cedars-Sinai Medical Center, Los Angeles, CA, USA

162. Medical Population Genetics, Broad Institute of Harvard and MIT, 5 Cambridge Center, Cambridge MA 02142, USA

163. National Heart, Lung and Blood Institute and its Framingham Heart Study, 73 Mount Wayte Ave., Suite #2, Framingham, MA 01702, USA

164. Department of Neurology and Medicine, University of Washington, Seattle, USA

165. Department of Medicine (Geriatrics), University of Mississippi Medical Center, Jackson, MS, USA

166. Department of Neurology, Boston University School of Medicine, USA

167. Finnish Institute of Occupational Health, Finnish Institute of Occupational Health, Aapistie 1, 90220 Oulu, Finland

168. Wellcome Trust Centre for Human Genetics, University of Oxford, UK

169. Lapland Central Hospital, Department of Physiatrics, Box 8041, 96101 Rovaniemi, Finland

170. Center for Non-Communicable Diseases Karachi, Pakistan

171. Atherosclerosis Research Unit, Department of Medicine, Karolinska Institute, Stockholm, Sweden

172. Department of Internal Medicine, University Medical Center Groningen, University of Groningen, The Netherlands

173. Department of Medical Genetics, Erasmus Medical Center, Rotterdam, The Netherlands

174. Gerontology Research Center, National Institute on Aging, Baltimore, MD 21224, USA

175. Istituto di Neurogenetica e Neurofarmacologia, Consiglio Nazionale delle Ricerche, Cittadella Universitaria di Monserrato, Monserrato, Cagliari, Italy

176. Unita` Operativa Semplice Cardiologia, Divisione di Medicina, Presidio Ospedaliero Santa Barbara, Iglesias, Italy

177. Computational Medicine Research Group, Institute of Clinical Medicine, University of Oulu and Biocenter Oulu, 90014 University of Oulu, Oulu, Finland

178. NMR Metabonomics Laboratory, Department of Biosciences, University of Eastern Finland, 70211 Kuopio, Finland

179. Department of Internal Medicine and Biocenter Oulu, Clinical Research Center, 90014 University of Oulu, Oulu, Finland

180. Institute for Molecular Medicine Finland FIMM, 00014 University of Helsinki, Helsinki, Finland

181. Department of Biomedical Engineering and Computational Science, School of Science and Technology, Aalto University, 00076 Aalto, Espoo, Finland

182. NUS Graduate School for Integrative Sciences & Engineering (NGS) Centre for Life Sciences (CeLS), Singapore, 117456, Singapore

183. Department of Internal Medicine B, Ernst-Moritz-Arndt-University Greifswald, 17487 Greifswald, Germany

184. Institute of Pharmacology, Ernst-Moritz-Arndt-University Greifswald, 17487 Greifswald, Germany

185. Institute for Community Medicine, Ernst-Moritz-Arndt-University Greifswald, 17487 Greifswald, Germany

186. U557 Institut National de la Santé et de la Recherche Médicale, U1125 Institut National de la Recherche Agronomique, Université Paris 13, Bobigny, France

187. Department of Neurology, Mayo Clinic, Jacksonville, FL, USA

188. Imperial College Cerebrovascular Unit (ICCRU), Imperial College, London, W6 8RF, UK

189. Faculty of Medicine, University of Split, Croatia

190. Department of Internal Medicine, Diabetology, and Nephrology, Medical University of Silesia, 41-800, Zabrze, Poland

191. Public Health Sciences section, Division of Community Health Sciences, University of Edinburgh, Medical School, Teviot Place, Edinburgh, EH8 9AG, UK

192. School of Science and Engineering, University of Ballarat, 3353 Ballarat, Australia

193. Prevention and Care of Diabetes, Department of Medicine III, Medical Faculty Carl Gustav Carus at the Technical University of Dresden, 01307 Dresden, Germany

194. University Hospital Münster, Internal Medicine D, Münster, Germany

195. Department of Medical Statistics, Epidemiology and Medical Informatics, Andrija Stampar School of Public Health, University of Zagreb, Croatia

196. AstraZeneca R&D, 431 83 Mölndal, Sweden

197. Genetic Epidemiology & Biostatistics Platform, Ontario Institute for Cancer Research, Toronto

198. Samuel Lunenfeld Institute for Medical Research, University of Toronto, Canada

199. Faculty of Epidemiology and Population Health, London School of Hygiene and Tropical Medicine, UK

200. Department of Epidemiology and Public Health, Imperial College, Norfolk Place London W2 1PG, UK

202. Department of Internal Medicine, Division of Cardiovascular Medicine, University of Michigan Medical Center, Ann Arbor, Michigan, USA

203. Singapore Eye Research Institute, Singapore, 168751, Singapore

204. Department of Ophthalmology, National University of Singapore, Singapore, 119074, Singapore

205. Department of Medicine, Yong Loo Lin School of Medicine, National University of Singapore, Singapore, 119074, Singapore

206. Duke-National University of Singapore Graduate Medical School, Singapore, 169857, Singapore

207. Division of Biostatistics, Washington University School of Medicine, Saint Louis, MO, 63110, USA

208. Department of Primary Care & Population Health, UCL, London, UK, NW3 2PF, UK

209. BHF Glasgow Cardiovascular Research Centre, University of Glasgow, 126 University Place, Glasgow, G12 8TA, UK

210. Epidemiology Public Health, UCL, London, UK, WC1E 6BT, UK

211. Departments of Epidemiology, Biostatistics, and Medicine, Johns Hopkins University, Baltimore MD, USA

212. Division of Nephrology, Department of Internal Medicine, University Medical Center Groningen, University of Groningen, The Netherlands

213. Division of Cardiology, Brigham and Women's Hospital, 900 Commonwealth Avenue East, Boston MA 02215, USA

214. Geriatric Rehabilitation Unit, Azienda Sanitaria Firenze (ASF), Florence, Italy

215. National Institute for Health and Welfare, Diabetes Prevention Unit, 00271 Helsinki, Finland

216. Hjelt Institute, Department of Public Health, University of Helsinki, 00014 Helsinki, Finland

217. South Ostrobothnia Central Hospital, 60220 Seinäjoki, Finland

218. Red RECAVA Grupo RD06/0014/0015, Hospital Universitario La Paz, 28046 Madrid, Spain

219. Department of Neurology, General Central Hospital, 39100 Bolzano, Italy

220. CIBER Epidemiología y Salud Pública, 08003 Barcelona

221. Department of Medicine and Department of Genetics, Harvard Medical School, Boston, Massachusetts 02115, USA

222. Geriatric Research and Education Clinical Center, Veterans Administration Medical Center, Baltimore, MD, USA

223. U872 Institut National de la Santé et de la Recherche Médicale, Centre de Recherche des Cordeliers, Paris, France

224. Institute of Physiology, Ernst-Moritz-Arndt-University Greifswald, 17487 Greifswald, Germany

225. Institute of Clinical Medicine/Obstetrics and Gynecology, University of Oulu, Finland

226. Service of Medical Genetics, Centre Hospitalier Universitaire Vaudois, 1011 Lausanne, Switzerland

227. Human Genetics Center, 1200 Hermann Pressler, Suite E447 Houston, TX 77030, USA

228. Division of Epidemiology and Prevention, Boston University School of Medicine, Boston, MA, USA

229. Department of Mathematics, Boston University, Boston, MA, USA

233. MRC-HPA Centre for Environment and Health, School of Public Health, Imperial College London, Norfolk Place, London W2 1PG, UK

234. Centre of Medical Systems Biology (CMSB 1-2), NGI Erasmus Medical Center, Rotterdam, The Netherlands
